# Anterior segment and external ocular disorders associated with HIV infections in the era of HAART in Chiang Mai University Hospital, a prospective descriptive cross sectional study

**DOI:** 10.1371/journal.pone.0193161

**Published:** 2018-02-21

**Authors:** Tassapol Singalavanija, Somsanguan Ausayakhun, Chulaluck Tangmonkongvoragul

**Affiliations:** Ophthalmology department, Chiang Mai University Hospital, Chiang Mai, Thailand; Boston University School of Medicine, UNITED STATES

## Abstract

Human immunodeficiency virus (HIV) causes impairment to the human immune system which leads to immunocompromised conditions, including ocular complications. Several important HIV-associated disorders may involve the anterior segment, ocular surface, and adnexae organ such as dry eye, blepharitis which reduce quality of life of patients. In present, potent antiretroviral therapies HAART (highly active antiretroviral therapy) has improved the length and quality of life which may lead to an increased prevalence of anterior segment ocular disorders. Hence, this study has been undertaken to identify the prevalence and associated factors of anterior segment and external ocular disorder in HIV infected patients in the era of HAART. A prospective descriptive cross sectional study was carried out in HIV positive patients conducted at the Department of Ophthalmology, Chiang Mai University Hospital, from February 2014 to October 2015. Detail history and ocular examination was carried out to examine for anterior segment and external ocular disorders. A total number of 363 patients were included for this prospective cross-sectional study. From the total of 363 patients, 123 patients had an anterior segment and external ocular disorder which account as the prevalence of 33.9%. The most common anterior segment manifestations was dry eye seen in 36 patients (9.9%), followed by posterior blepharitis (Meibomian gland dysfunction) seen in 23 patients (6.3%) and anterior blepharitis seen in 12 patients (3.3%). Other ocular complications included microvasculopathy, immune recovery uveitis, conjunctivitis, papilloma, anterior uveitis, corneal ulcer, nevus, trichiasis, molluscum contangiosum, Kaposi sarcoma, interstitial keratitis, conjunctival lymphangiectasia, dacryocystitis, vernal keratoconjunctivitis and eyelid penicilosis. In this study, the prevalance of anterior segment disorders was higher than in the preHAART era. Dry eye, blepharitis and uveitis were the top three most common anterior segment disorders in the HAART era. The statistical analysis showed no association between age, sex, CD4 count, duration of infection or receiving HAART and anterior segment disorders. Anterior segment abnormalities reduce the quality of life of patients, so ophthalmologists have to be aware and complete ocular examination should be performed in all HIV infected patients.

## Introduction

Nowadays, Human Immunodeficiency Virus (HIV) is still a major public health problem in Thailand. Even though there are many campaigns, raising the importance of HIV prevention, Thailand AIDS’s response program report in 2012 shows that the number of newly diagnosed HIV patients is still increasing. [1] The majority groups are especially female sex workers, men who have sex with men (MSM), and people who use drugs [2,3].

The surveillance for HIV infection from the division of epidemiology, The Ministry of Public Health of Thailand from September 1984 to March 2011 revealed that the number of HIV infected patients in Thailand was 371,844 in which 114,043 patients were in the northern part of Thailand. [2,3]

HIV causes impairment to the human immune system which leads to immunocompromised conditions and allows for opportunistic infections to invade many organs, including the eye. Due to the fact that cytomegalovirus retinitis is the most prevalent cause of visual impairment in individuals with the acquired immunodeficiency syndrome (AIDS)[4], many researchers have focused primarily on the posterior segment examination. However, several HIV-associated disorders may involve the anterior segment, ocular surface, and adnexae organ, such as keratoconjunctivitis sicca and blepharitis. At present, the introduction of potent antiretroviral therapies (known as Highly Active Antiretroviral Therapy or HAART) has dramatically changed the prevalence of the AIDS epidemic. Poorer availability of HAART in most developing countries is reflected by a higher level of profound immune suppression, with increased susceptibility to opportunistic infections.[5] During the HAART era, there has been an estimated 80% decrease in the incidence of CMV retinitis.[6] With HAART, there is a reduction in the number of opportunistic infections, improvement in the length and quality of life; patients are less likely to be blind from posterior segment infections.[7] This longer survival rate may lead to an increased prevalence of the anterior segment and external ocular disorders. Therefore, anterior segment and external ocular disorders become greater concern in the HAART era by affecting the quality of life of infected individuals. Hence, this study has been undertaken to identify the prevalence of the anterior segment and external ocular disorders in HIV infected patients in Chiang Mai University Hospital and to find the associated factors of the abnormal manifestations.

## Materials and methods

The study was a prospective cross sectional study, conducted at the department of Ophthalmology, Chiang Mai University Hospital, a tertiary health care center and teaching hospital in northern Thailand. The recruitment population consisted of HIV-positive patients diagnosed by the methods of DAGS ELISA, Combi ELISA, and Rapid Test (qualitative immunochromatography assay) at the outpatient department, ocular infectious unit, CMV (Cytomegalovirus) retinitis clinic and the infectious unit of medicine between February 2014 and May 2015. This study was reviewed and approved by the institutional review board, Chiang Mai University. Written and verbal consents were obtained from the patients. The patient was informed for the purpose, the details of the research and must participate in a voluntary way. The patient has to sign for the informed consent document. In a child under 13 years old, the written document was signed for permission by the parents. The institutional review board approved for the consent procedure and all documents. Detail history including demographic data of age, gender, duration of HIV infection, type of HAART therapy, recent CD4 T-cell count level and associated systemic symptoms were recorded. After that, patients were evaluated by the dry eye questionnaire, which has been used by Schein et al. [8] Detailed ocular examination was carried out by slit lamp biomicroscopy. Visual acuity assessment, external adnexal organ and anterior segment were examined. Any abnormal findings of eyelid, orbital region, conjunctiva, cornea, anterior chamber cells and flare grading, sclera and episclera were recorded. The dry eye diagnosis criteria included both of subjective discomfort symptoms (if one or more symptoms were noted as severe from the questionnaire) and clinical signs of ocular surface disorder such as punctate epithelial erosion or tear break up time less than 10 seconds. Conjunctival microvasculopathy was characterized by microaneurysm formation, segmental capillary dilatation and constriction, and aberrant curved vessels.[9] The anterior uveitis defined as an inflammation of the iris and ciliary body with inflammatory cells in the anterior chamber. Immune recovery uveitis was diagnosed when noninfectious intraocular inflammation developed in patients with inactive CMV retinitis who showed immune response with HAART. Immune recovery is defined as an increase in CD4 T cell count from 50 cells/μL or more to a level of 100 cells/μL or more.[10]

The data was analyzed with descriptive statistics analysis. Univariable analyses were performed with SPSS version 16.0. Correlation between anterior segment abnormalities and associated factors were determined by binary logistic regression. Multiple logistic regression was used to adjust for confounding factors. The strength of association was measured using Odds ratio and its 95% confidence interval. The P value of < 0.05 was considered as statistically significant.

## Results

A total number of 363 patients were included in this study. The age of the patients ranged from 9 to 72 years, the majority of patients were in the age group of 40–49 years. The total number of affected patients were 174 males (47.9%) and 189 females (52.1%). The average duration of HIV infection was 6.86 (±6.27) years. The average of the CD4 T-cell count was 361.86 ± 283. 97.2% of these patients (353/363) were receiving HAART. The reasons for those who did not receive anti-retroviral medication mostly were new cases with first diagnosis. One patient was infected with pulmonary tuberculosis, which resulted in the delay of the antiretroviral medication. There is no significant difference in gender and ages between the patients with and without anterior segment complications (X2test, P > 0.05).

(Table 1)

**Table 1:** Baseline demographic and laboratory characteristics of 363 HIV infected patients.

Demographic data	Anterior segment manifestation		Non-Anterior segment manifestation		Total		P value
	N	%	N	%	N	%	
**Male**	59	16.3	115	31.7	174	47.9	0.993
**Female**	64	17.6	125	34.4	189	52.1	
**Average age**	41.83 ± 10.76		44.17 ± 11.51		42.62 ± 11.06		0.056
Less than 10	1	0.3	0	0	1	0.3	0.335
10–19	0	0	6	1.6	6	1.6	
20–29	9	2.5	22	6.1	31	8.6	
30–39	36	9.9	66	18.2	102	28.2	
40–49	43	11.9	89	24.6	132	36.5	
50–59	19	5.2	43	11.9	62	17.1	
60 or more	15	4.1	14	3.9	29	8.0	
**CS4+ counts (cells/μL)**	343.08 ± 278		398.04 ± 298		361.86 ± 283		0.082
**Duration of infection (years) (Median± IOR25,75)**	5 (1–10)		8 (2–12)		5 (1–10)		0.003
**On HAART**	119	32.8	234	64.5	353	97.2	0.739
**Not on HAART**	4	1.1	6	1.7	10	2.8	

**Fig 1:**
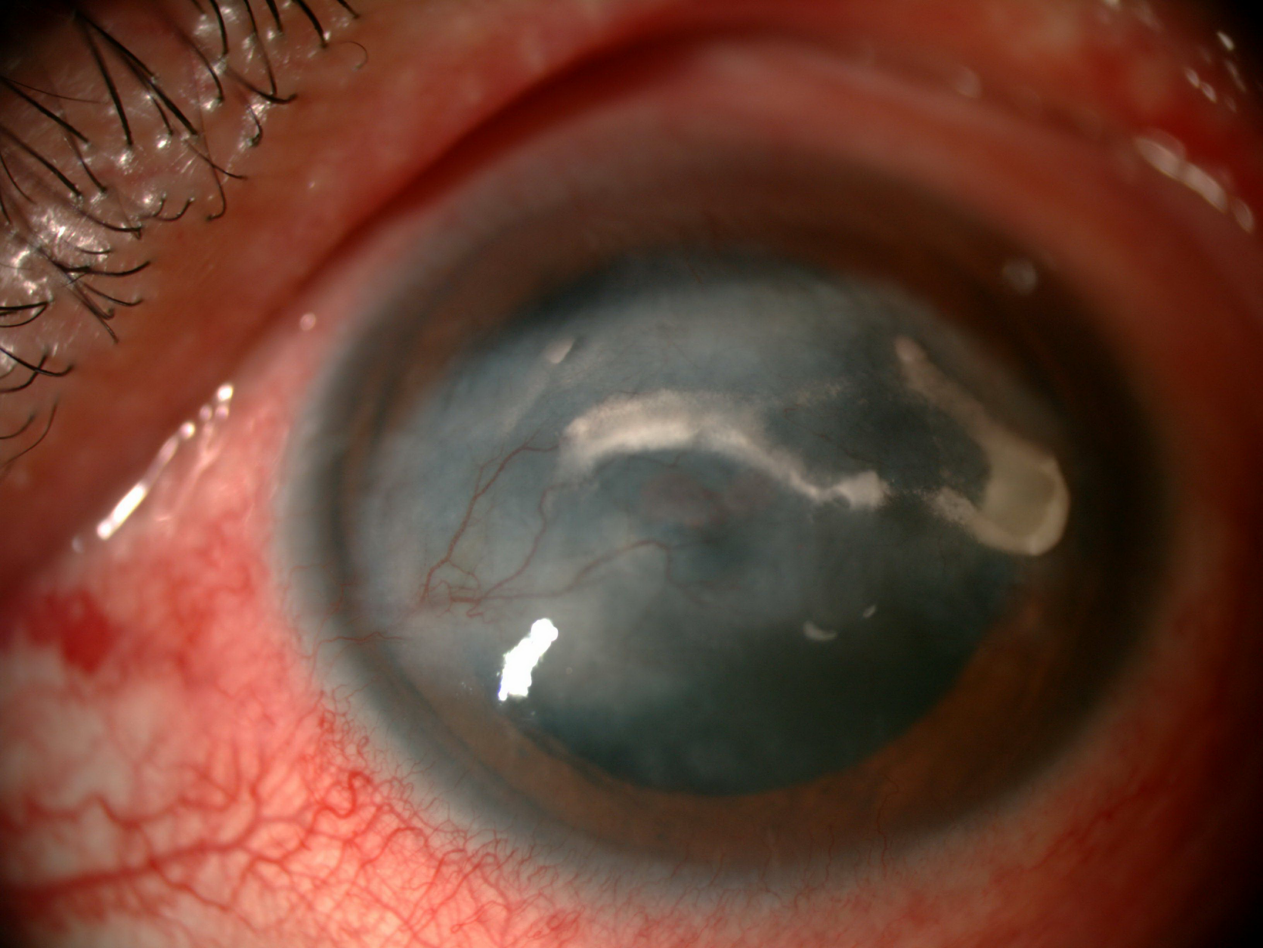
Severe dry eye with lipid deposit.

**Fig 2:**
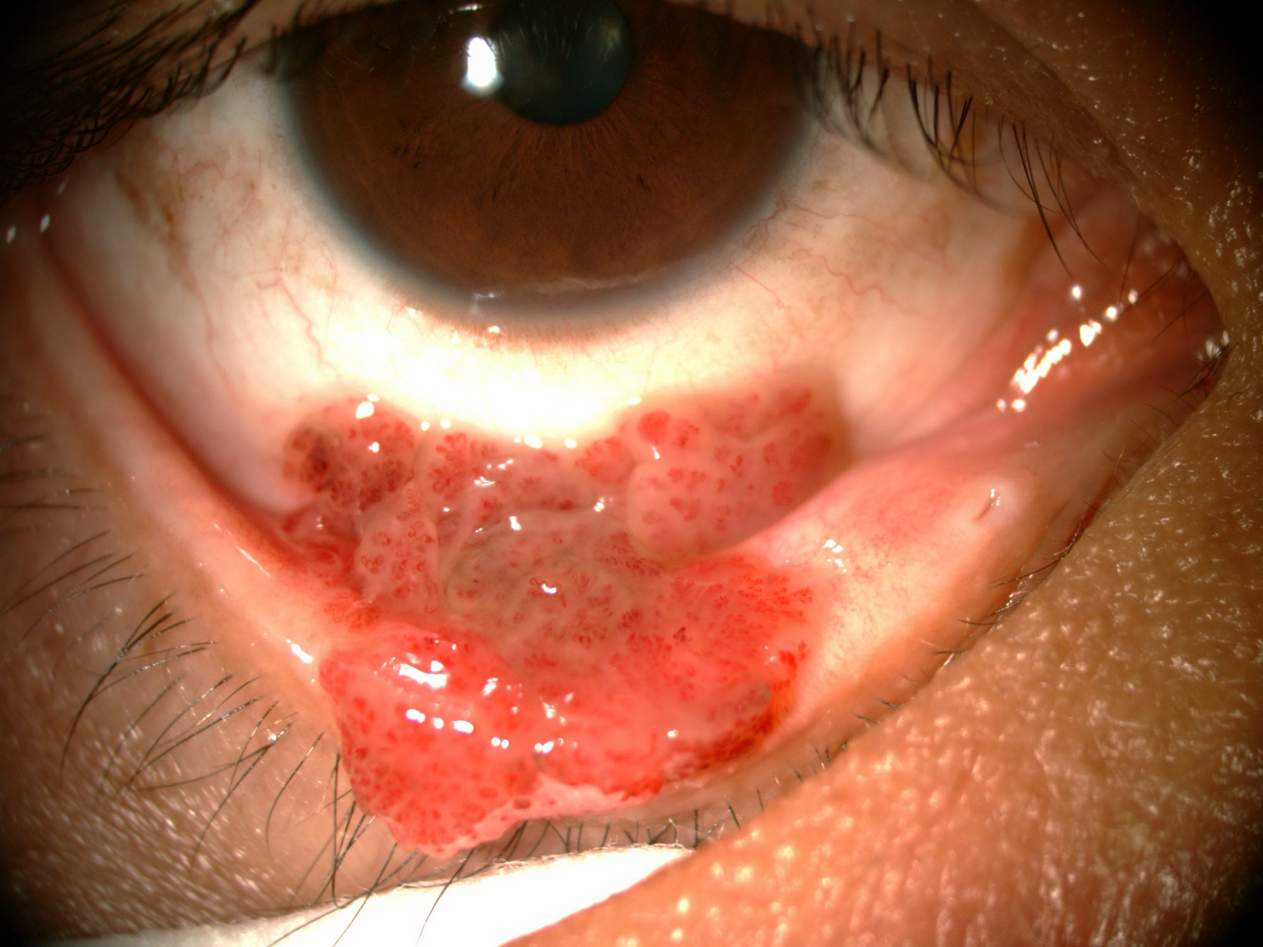
Papilloma.

**Fig 3:**
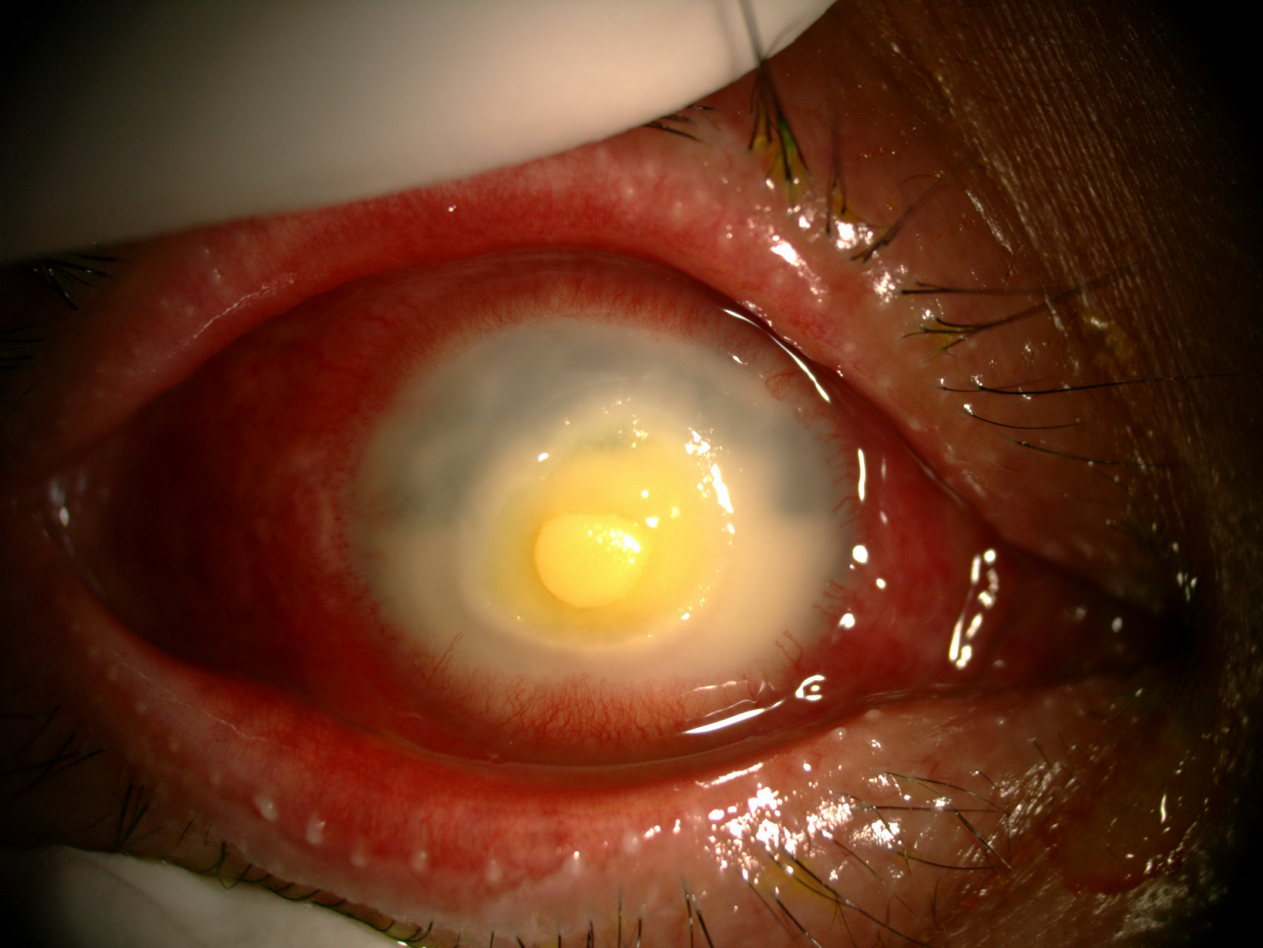
Severe corneal ulcer with perforation.

**Fig 4:**
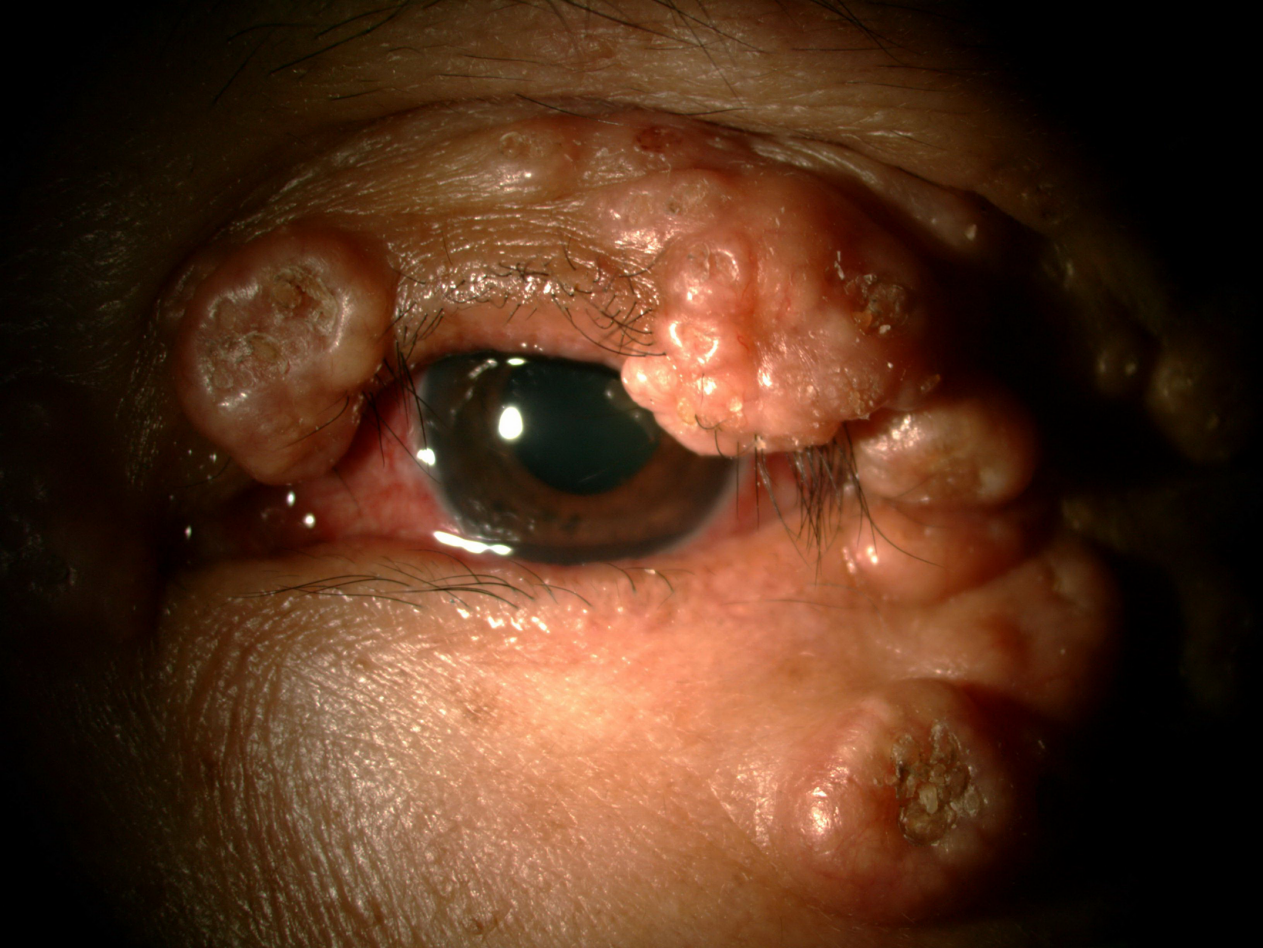
Severe molluscum contagiosum.

**Fig 5:**
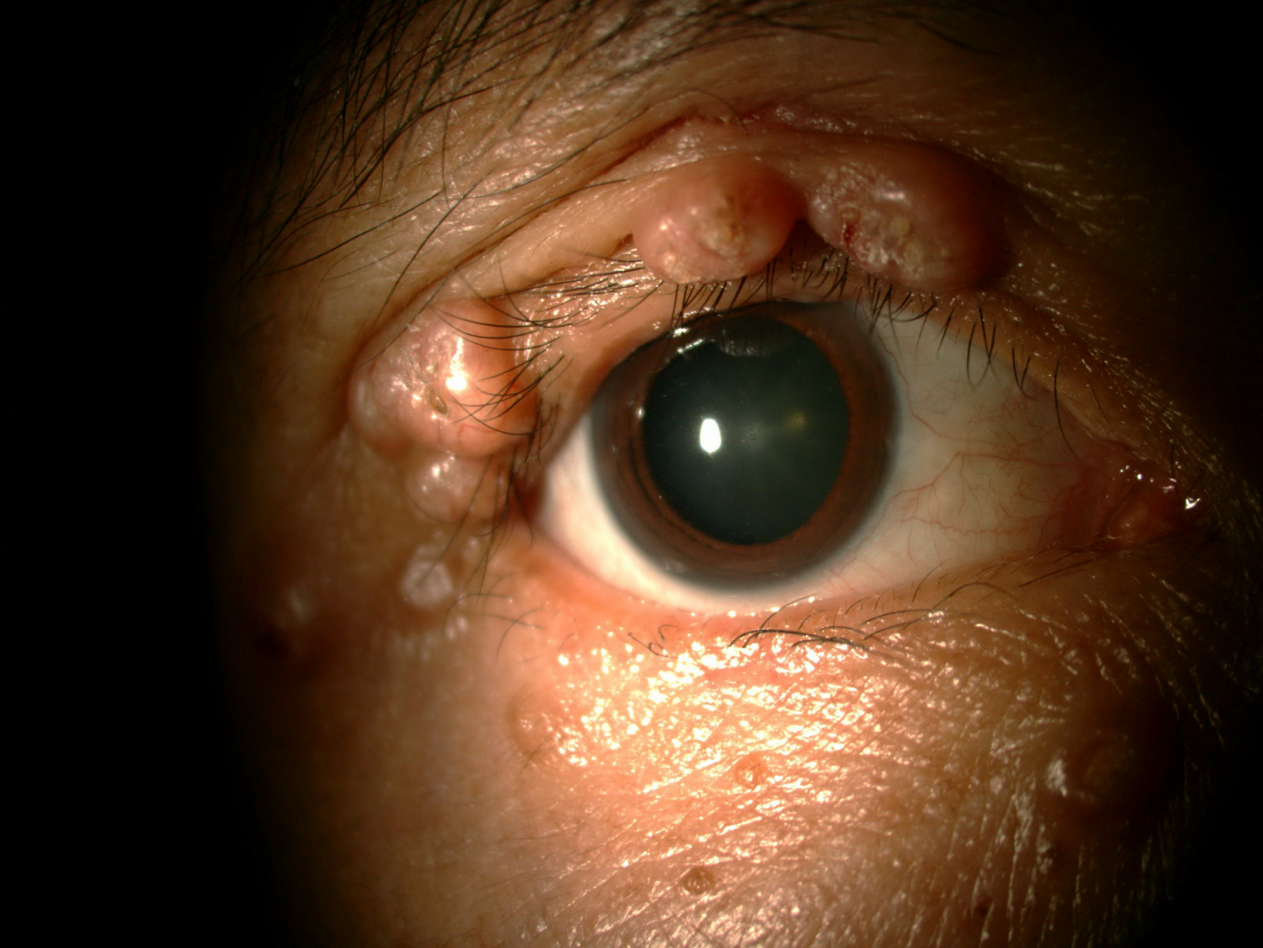
Severe molluscum contagiosum.

**Fig 6:**
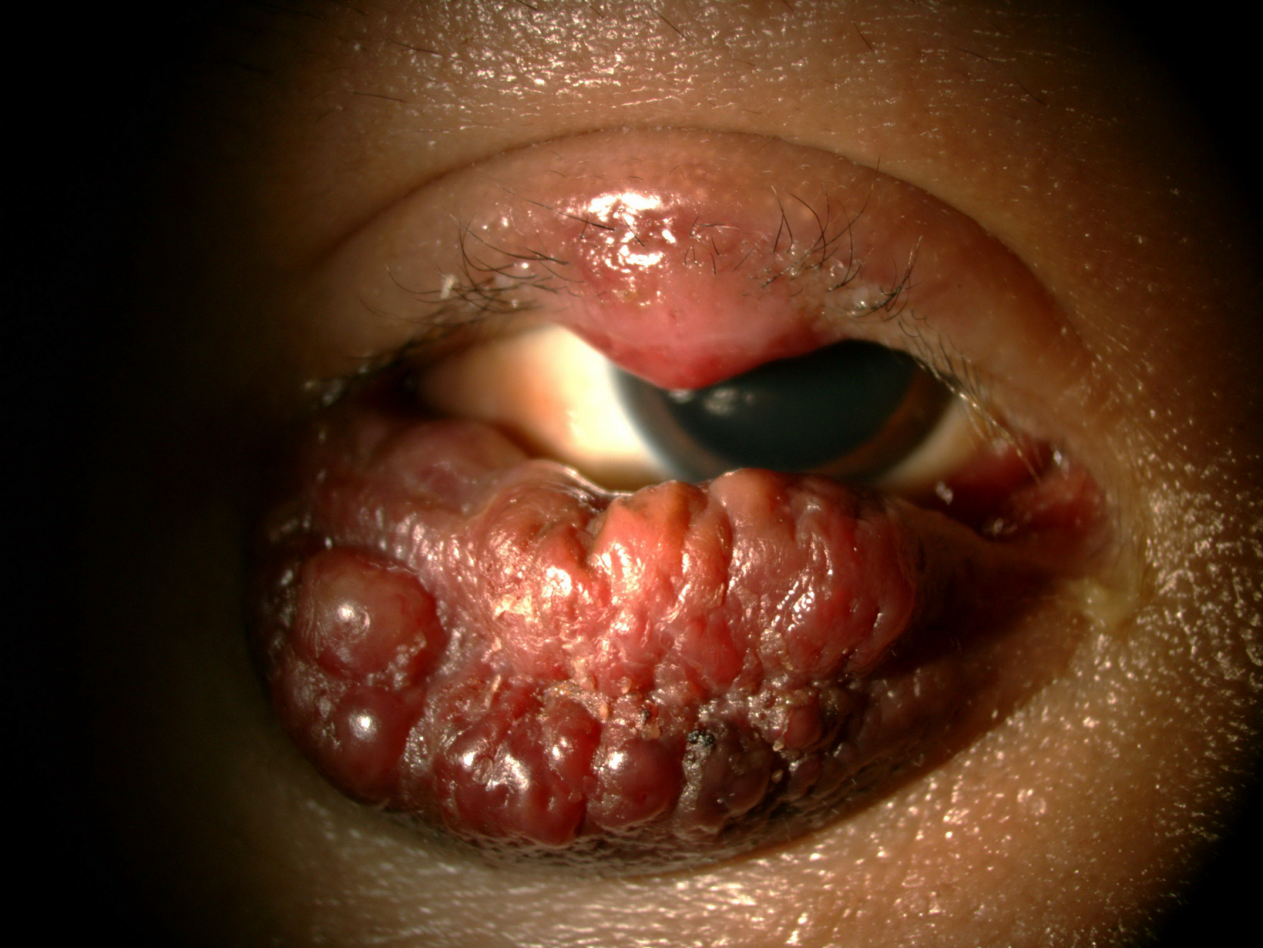
Kaposi sarcoma.

**Fig 7:**
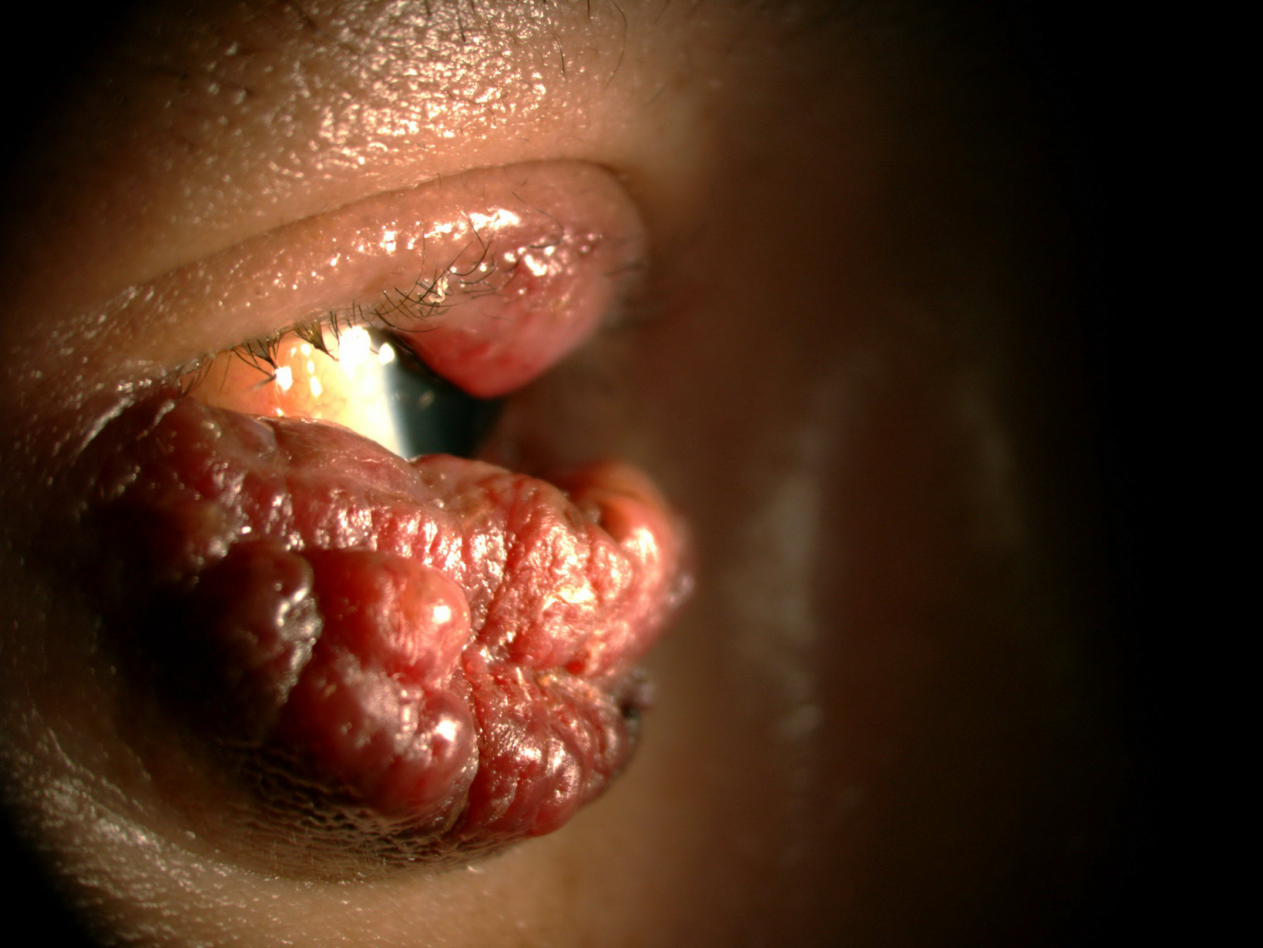
Kaposi sarcoma.

The prevalence of anterior segment and external ocular disorder was 33.9% (123 out of 363) with 95% CI = 0.289–0.386. Table 2 showed prevalence, mean CD4 level of anterior segment and external ocular disorder. The most common anterior segment manifestation was dry eye seen in 36 patients (9.9%). There were wide ranges of severity of dry eye including severe dry eye with lipid deposit on ocular surface (Fig 1) to moderate dry eye with symptom of discomfort with punctate epithelial erosion. The second and third anterior segment disorders were posterior blepharitis or Meibomian gland dysfunction, 23 patients (6.3%) and anterior blepharitis, 12 patients (3.3%) respectively. There were 9 cases (2.5%) of microvasculopathy, 9 cases (2.5%) of immune recovey uveitis, 4 (1.1%) cases of anterior uveitis, 6 cases (1.7%) with conjunctivitis, 6 cases (1.7%) with papilloma (Fig 2) and 2 cases (0.6%) with trichiasis. Four cases (1.1%) of corneal ulcer were found in this study. The first case presented was fungal corneal ulcer. The second case presented with severe total corneal ulcer which could not identify organism on corneal scraping. The third case was a corneal ulcer which developed subsequently from severe dry eye which responded to antibiotic treatment The last case presented with severe corneal ulcer with 4 mm of hypopyon and nearly total corneal infiltration (Fig 3), corneal scraping showed no organism, finally evisceration was advised. Other anterior segment and external adnexal disorders were 3 cases (0.8%) of nevus, 2 cases (0.6%) of molluscum contangiosum (Figs 4 and 5) and each case of Kaposi sarcoma (Figs 6 and 7), interstitial keratitis, conjunctival lymphangiectasia, dacryocystitis, vernal keratoconjunctivitis and eyelid penicilosis. One case of molluscum contangiosum presented with multiple large, round, centrally umbilicated pearly papules on the eyelid and periorbital skin. The CD4 level at the time of presentation was 27 cells/μl and the patient received an HARRT for about one week before the referral. In a Kaposi sarcoma case, the patient presented with progressive enlargement of irregular lower lid mass which responded to the treatment of chemotherapy (Paclitaxel) for 5 cycles followed by IFRT (involved-field radiation therapy). The other 240 patients (66.1%) showed no anterior segment and ocular adnexal abnormality.

**Table 2:** Prevalence and mean CD4 level of each types of anterior segment and external ocular disorders.

Ocular disorder	CD4 T-cell level		Mean CD4 (±SD)	Number of cases	Number of eyes	Overall prevalence
	≤ 200	>200				
No ocular disorder	88(24.4%)	150 (41.6%)	343.04 (±278.04)	241	482	66.3%
Dry eye	7 (1.9%)	29 (8%)	486.83 (±283.06)	36	68	9.9%
Posterior blepharitis	6 (1.7%)	17 (4.7%)	556.78 (±316.45)	23	46	6.3%
Anterior blepharitis	2 (0.6%)	10 (2.8%)	444.25 (±223.46)	12	21	3.3%
Conjunctival microvasculopathy	0	9 (2.5%)	567.11 (±225.16)	9	14	2.5%
Immune recovery uveitis	5 (1.4%)	4 (1.1%)	117.89 (±101.57)	9	15	2.5%
Conjunctivitis	3 (0.8%)	3 (0.8%)	356.50 (±262.68)	6	11	1.7%
Papilloma	1 (0.3%)	5 (1.4%)	522.50 (±273.48)	6	7	1.7%
Anterior uveitis	3 (0.9%)	1 (0.3%)	109.50 (±117.72)	4	6	1.1%
Corneal ulcer	1 (0.3%)	3 (0.8%)	439.25 (±276.55)	4	4	1.1%
Nevus	1 (0.3%)	2 (0.6%)	451.00 (±371.58)	3	4	0.8%
Trichiasis	0	2 (0.6%)	810.00 (±475.18)	2	3	0.6%
Molluscum contangiosum	2 (0.6%)	0	63.50 (±51.61)	2	2	0.6%
Kaposi sarcoma	1 (0.3%)	0	19	1	1	0.3%
Interstitial keratitis	0	1 (0.3%)	432	1	1	0.3%
Conjunctival lymphangiectasia	0	1 (0.3%)	288	1	1	0.3%
Dacryocystitis	0	1 (0.3%)	235	1	1	0.3%
Vernal keratoconjunctivitis	1 (0.3%)	0	17	1	2	0.3%
Eyelid penicilosis	0	0	7	1	2	0.3%

Upon analysis of the correlation between anterior segment disorders and associated factors. Multivariate analysis showed no correlation between sex, age, CD4 count, receiving HARRT or duration of infection and anterior segment disorders. (Table 3).

**Table 3:** Multivariate analysis of associated factors for the anterior segment and external ocular disorders in human immunodeficiency virus-infected patients.

Associated factor		Ocular manifestation (%)				Unadjusted OR (95% CI)	Adjusted OR (95% CI)	P-value (Adjust OR)
		Yes		No				
		N	%	N	%			
Sex ^§^	Male Female	59 64	16.3 17.6	115 125	31.7 34.4	0.998 (0.646–1.542)	0.935 (0.594–1.472)	0.772
Age (yr)^ɸ^	≤ 40 > 40	55 69	14.9 19.1	104 135	28.7 37.3	1.016 (0.655–1.575)	1.202 (0.748–1.931)	0.446
CD4+ count ^ʈ^ (cells/ μL)	< 200 ≥ 200	44 81	11.6 22.4	88 150	24.4 41.6	0.884 (0.562–1.395)	1.035 (0.613–1.747)	0.899
HAART ^‡^	Yes No	119 4	32.8 1.1	234 6	64.5 1.7	1.311 (0.363–4.735)	1.616 (0.424–6.162)	0.482
Duration of infection (yr)^α^	< 5 ≥ 5	44 79	35.8 64.2	119 121	49.6 50.4	1.766 (1.129–2.761)	1.916 (1.152–3.187)	0.062

Values are presented as number (%); Adjusted for sex, age, CD4+ count, HAART and duration of infection.

OR = odds ratio; CI = confidence interval; HAART = highly active antiretroviral therapy

^§^; adjusted for age, CD4+ cell count, HAART and duration of infection

^ɸ^; adjusted for sex, CD4+ cell count, HAART and duration of infection

^ʈ^; adjusted for age, sex, HAART and duration of infection

^‡^; adjusted for age, sex, CD4+ cells count and duration of infection

^α^; adjusted for age, sex, CD4+ cells count and HAART

## Discussion

In this study, a total of 123 patients (33.9%) had various anterior segment and external ocular manifestations. Although patients receiving HAART reduce the risk of opportunistic infections and recover the immune function, the prevalence of anterior segment disorders in this study (33.9%) was still high compared to the pre HAART era reported by Ausayakhun et al. (8%) in 2003.[11] The report by Ausayakhun et al., epidemiology of the ocular complications of HIV infection in Chiang Mai 2003, of the 395 HIV positive patients, only 21 patients (0.05%) received antiretroviral drugs comparing to our study which 353 from 363 cases (97.2%) received these drugs.

Many studies showed the prevalence of ocular manifestation in HIV patients ranged from 37.7% to 75% [12–17], most of them did not include anterior segment manifestations which makes it difficult to compare to our study. In Asian countries, Joshi and associates from Nepal reported the prevalence of anterior segment involvement was 9.7% [18], while the report of Young Shin Kim from Korea was 28.3%.[15] The difference is likely due to the inclusion criteria and spectrum of ocular manifestations considered in each region were not the same.

The higher prevalence of anterior segment disorders in this study compared to the pre-HAART era could be explained by our focus on the anterior segment changes in HIV infected patients who presented with both ophthalmic and non-ophthalmic problems. Some asymptomatic ocular manifestations such as small papilloma, molluscum contagiosum and nevus were detected and increased the prevalence of this study. Another reason was a longer survival and a better quality of life after the introduction of HAART leading the patients to more concern about minor eye symptoms such as eye discomfort and dryness. The study from Young Shin Kim found that the introduction of HAART has changed the landscape of ocular presentations in patients with AIDS, the anterior segment and external ocular manifestations occurred more frequently than the posterior segment manifestations at 28.3% and 19.7%, respectively [19].

This study revealed that dry eye was the most common anterior segment disorder with the prevalence of 9.9% comparing to the previous report by S.Ausayakhun (2003) which was 2%.[11]. Keratoconjunctivitis sicca appears to be more common among individuals with AIDS (16.9% to 38.8%)[20–22]. Severe dry eye in HIV patients may relate to the possibility of an autoimmune-like pathogenesis of abnormalities of tear production which associated with lymphocytic infiltration and eventual destruction of the lacrimal gland acini and ducts.[23]

Blepharitis was generally found in this study. The pathogenesis of blepharitis in immunodeficient individuals may either involve a reduced ability to control normal flora or more complex changes in cutaneous glands of the eyelids that occur with immunosuppression [24]

The percentage of anterior uveitis of this study was 1.1% (4 cases). Mild iridocyclitis is common in HIV-positive patients and is usually observed in association with retinitis caused by cytomegalovirus [25] or varicella–zoster virus [26]. Low immune status caused an opportunistic infection to invade the body easily which caused inflammatory reaction and developed anterior uveitis.

Immune recovery uveitis (IRU) was found 2.5% in this study. IRU is a result of heightened immunological reactions against intraocular pathogens, usually cytomegalovirus (CMV), that become possible with immune reconstitution [27,28]. People with extensive CMV retinitis lesions are at increased risk for IRU, presumably because there is more viral antigen in the eye.[29]

Only two cases of extensive molluscum contagiosum lesion were found in this study, we believe that HIV attributes to the profound dysfunction of T-lymphocyte-mediated immune response which molluscum contagiosum more disseminate than normal population.

Neoplasm is not rare in HIV infected patients. While Kaposi sarcoma of the eyelids or conjunctiva affected up to 10% of HIV-infected patients [30], We found only one Kaposi sarcoma on eyelid. This case was confirmed by the tissue biopsy and the patient improved spontaneously after receiving both chemotherapy and radiation therapy.

Other anterior segment and external ocular disorders in this study were conjunctival microvasculopathy, conjunctivitis, papilloma, trichiasis, corneal ulcer, nevus, interstitial keratitis, conjunctival lymphangiectasia, dacryocystitis, vernal keratoconjunctivitis, and eyelid penicillosis.

In data analysis, we did not find any associated factors related to the anterior segment and external ocular disorders.

The limitation of our study were small number of each type of anterior segment abnormality which made the associated factors cannot be calculated. We were unable to obtain tissue biopsies from all conjunctival or skin lesions but the clinical diagnosis was made by agreement of all investigators included in the study.

## Conclusions

In the era of HAART, there were improvements in survival and quality of life of HIV-infected patients. The incidence of sight-threatening complications has decreased, but the anterior segment and external ocular disorders were still commonly found. This study showed the prevalence of the anterior segment disorders as high as 33.9%. Dry eye, blepharitis and uveitis were the top three most common anterior segment disorders in the HAART era. Comparing to preHAART, the prevalence was 8% and the most common anterior segment disorder were uveitis, dry eye and keratitis respectively. The anterior segment abnormality may reduce the quality of life of patients, so ophthalmologists have to be aware and complete ocular examination should be performed in all HIV infected patients.
